# Hydrogen–Dislocation Interactions at Cryogenic Temperatures: Serrated Yielding and Embrittlement Resistance in High-Strength Austenitic Alloys

**DOI:** 10.3390/ma18225109

**Published:** 2025-11-10

**Authors:** Nina Damm, Marina Lukas, Jan Platl, Andreas Drexler, Matthias Eichinger, Magdalena Eskinja, Gregor Mori, Zoltán Simon, Michael Scheerer, Stefan Marsoner, Vsevolod I. Razumovskiy

**Affiliations:** 1 Materials Center Leoben Forschung GmbH, Vordernberger Straße 12, 8700 Leoben, Austriavsevolod.razumovskiy@mcl.at (V.I.R.); 2 voestalpine BÖHLER Edelstahl GmbH & Co KG, Mariazeller Straße 25, 8605 Kapfenberg, Austriaandreas.drexler@bohler-edelstahl.at (A.D.); 3 Chair of General and Analytical Chemistry, Montanuniversität Leoben, Franz-Josef-Straße 18, 8700 Leoben, Austria; 4 Aerospace & Advanced Composites GmbH, Viktor-Kaplan-Straße 2, Building F, 2700 Wiener Neustadt, Austria; zoltan.simon@aac-research.at (Z.S.); michael.scheerer@aac-research.at (M.S.)

**Keywords:** hydrogen embrittlement, cryogenic temperature, liquid helium temperature, serrated yielding, hydrogen-dislocation interaction

## Abstract

Comprehensive studies of hydrogen embrittlement in high-strength austenitic alloys under cryogenic conditions are scarce, leaving the combined effect of hydrogen charging and extreme temperatures largely unexplored. Given the demands of cryogenic applications such as hydrogen storage and transport, understanding material behavior under these conditions is crucial. Here, we present the first systematic study of hydrogen’s effect at liquid helium temperature (4.2 K) on the mechanical properties of precipitation hardened austenitic alloys, specifically the nickel-based Alloy 718 and austenitic stainless steel A286. Both materials were subjected to pressurized hydrogen charging at 473 K followed by slow strain rate tensile testing at room temperature and at 4.2 K. Hydrogen charging caused significant ductility loss at room temperature in both alloys. In contrast, testing at 4.2 K resulted in increased strength and no evidence of hydrogen embrittlement. Notably, materials pre-charged with hydrogen and tested at 4.2 K exhibited higher stress drop amplitudes and increased strain accumulation during serration events, suggesting persistent hydrogen–dislocation interactions and possible enhanced dislocation pinning by obstacles such as Lomer–Cottrell locks. These results indicate that while hydrogen influences plasticity mechanisms at cryogenic temperatures, embrittlement is suppressed, providing new insight into the safe development of austenitic alloys in cryogenic hydrogen environments.

## 1. Introduction

Nickel-based alloys and austenitic stainless steels feature a unique combination of properties, such as high strength, high ductility and excellent corrosion resistance, explaining their widespread use under extreme conditions [1,2]. However, when exposed to hydrogen-containing environments, these alloys may exhibit significant ductility loss caused by hydrogen embrittlement (HE) [3,4,5,6,7,8,9,10]. A notable example is the significant ductility loss observed in austenitic stainless steels of the 3xx series when exposed to hydrogen-containing environments at temperatures up to approximately 200 K [11,12]. Therefore, understanding the influence of temperature on HE is crucial for applications involving gaseous or liquid hydrogen tanks or valves operating at cryogenic temperatures [2].

The mechanical properties of nickel-based alloys and austenitic stainless steels have been studied extensively at cryogenic temperatures, where it has been shown that they typically remain ductile down to very low temperatures, even in combination with hydrogen [2,11,12,13,14,15,16,17,18,19,20]. Much less is known about precipitation hardened (PH) austenitic alloys [13,14,21], which are considered possible alternatives to conventional austenitic stainless steels for the renewable energy sector applications due to their superior strength [1]. The available literature’s data suggest that although Alloy 718 (UNS N07718) is relatively resistant to hydrogen ingress from the environment, there is a sizable effect of hydrogen on ductility loss at temperatures between 233 and about 573 K [13,14]. This is critical not only for hydrogen storage but also for aerospace, oil and gas and nuclear power applications, where it has been shown to depend not only on temperature but also on the hydrogen content in the material [14].

In this study, we focus on HE of the well-studied Alloy 718 and the austenitic stainless steel A286 (UNS S66286), both examined in their PH high-strength condition. Both alloys are strengthened by γ′ (Ni_3_(Al,Ti)) precipitates, while Alloy 718 is additionally reinforced by γ″ (Ni_3_Nb). Previous studies have reported that an increased γ″ fraction enhances susceptibility to HE [6]. Moreover, suppressing the formation of δ phase (Ni_3_Nb), the stable phase of γ″, has been shown to improve HE in Alloy 718 [6,8]. In contrast, A286 contains η phase (Ni_3_Ti), the stable phase of Ti rich γ′, which, when present along grain boundaries, correlates with higher HE susceptibility as its fraction increases [21]. Even in the absence of η phase, however, Takakuwa et al. [5] observed substantial ductility loss due to hydrogen pre-charging. Earlier studies on A286 also investigated the influence of lattice misfit on HE resistance [3,22] and identified carbides, γ′ and η phase as preferential crack initiation sites [4].

Alloy 718 and A286 in their PH state are among the most promising materials for applications in H-bearing environments at cryogenic temperatures. Typical cryogenic temperatures for HE testing addressed in the literature usually do not go below 77 K (boiling temperature of liquid nitrogen), though the operating temperature of hydrogen tanks and valves can reach 20 K or less [23].

At temperatures of 20 K and below, austenitic alloys show low temperature serrated deformation (LTSD) [24]. This behavior is typically governed by the (i) thermo-mechanical and (ii) mechanical effects. The former describes localized thermal softening due to local deformation that releases heat. This heat spike can reach very high values of several 10 to 100 K [24] and is very localized due to very low heat capacity and thermal conductivity at such temperatures [25]. This heat spike is thought to cause local thermal softening that causes avalanche shear of slip bands, which manifests in stress drops and causes characteristic serrations in a stress–strain curve. Alternatively, the mechanical effect describes dislocation accumulation in front of obstacles. Once a critical stress is reached, the piled-up dislocations are released, resulting in avalanche slip and a sudden proliferation event [26]. Although recent studies [26,27,28,29] investigated alloys differing in chemistry and precipitation state compared to the present work, their room temperature (RT) stacking fault energies (SFE) fall into the similar range of approximately 30 mJ/m^2^ [3]. Since SFE strongly governs dislocation mobility, partial separation and the likelihood of cross-slip, a similar mechanical response at cryogenic temperatures may be anticipated, which is consistent with a mechanical origin of the LTSD in the investigated alloys.

We investigate the mechanical properties of A286 and Alloy 718 at 4.2 K in both the hydrogen pre-charged und uncharged states. The details of this investigation and the differences in mechanical response of both alloys at RT and 4.2 K, with and without hydrogen pre-charging, are presented and discussed.

## 2. Materials and Methods

Alloy A286 and Alloy 718, with nominal compositions given in Table 1, were solution-annealed and subsequently aged to minimum yield strengths of 1034 MPa and 724 MPa at RT, respectively. Cylindrical tensile specimens with a diameter of 4 mm and a gauge length of 20 mm were machined from the centers of rolled bars with diameters of 31.75 mm for alloy A286 and 25.4 mm for Alloy 718. Their surface was ground to P2000 to ensure a fine surface finish and minimize the influence of machining marks on the results of the mechanical testing and hydrogen charging.

Hydrogen charging was performed to ensure sufficient uptake for significant ductility loss at ambient conditions. Half of the tensile specimens were charged with pressurized hydrogen at 473 K for 14 days at 1000 bar using the autoclave test bench described by Eichinger et al. [30]. This charging procedure excludes hydride formation, as Ni-H hydrides form only at pressures exceeding 10,000 bar at 473 K [31] and avoids niobium oxide formation at the on Alloy 718, which occurs only at temperatures of 800 K or higher [32]. Effective hydrogen diffusion coefficients at 473 K were extrapolated from literature values as 3.5 × 10^−8^ cm^2^/s for A286 [33] and 6.0 × 10^−8^ cm^2^/s for Alloy 718 [34,35]. Using these values, the hydrogen saturation levels at the specimen center (cylinder, 4 mm diameter) after the specified charging duration were calculated numerically by solving Fick’s second law with appropriate boundary conditions. This resulted in estimated saturation levels of 93% for A286 and 100% for Alloy 718, indicating near-uniform hydrogen uptake in both alloys under the applied charging conditions.

Total hydrogen content within the specimen gauge length was measured by thermal desorption spectroscopy using a Bruker Galileo G8 (Billerica, MA, USA), yielding 89 wt. ppm for A286 and 107 wt. ppm for Alloy 718. These measurements were performed using a rapid heating protocol where specimens were heated to 1173 K as quickly as possible and held for 15 min, without a classical defined heating rate typically applied in thermal desorption spectroscopy. Thermal desorption spectroscopy was chosen for its superior detection limit compared to conventional hot extraction techniques. The focus was exclusively on total hydrogen quantification rather than desorption kinetics or hydrogen trapping. Specimens were subsequently stored in liquid nitrogen at 77 K and tested within one week.

**Figure 1:**
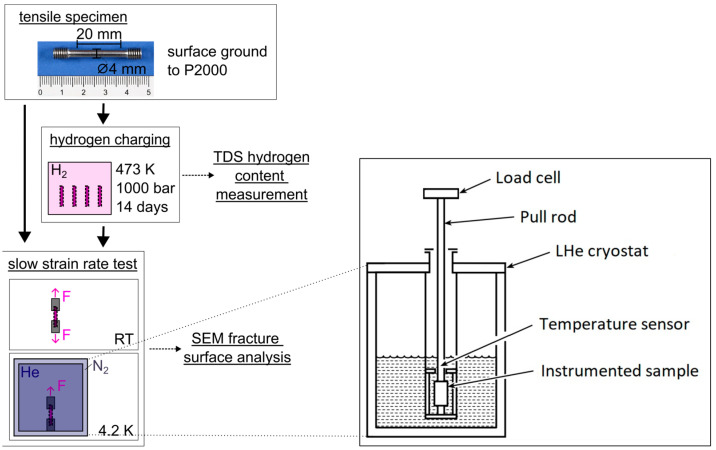
Schematic illustration of the mechanical testing setup. After manufacturing, half of the specimens were hydrogen charged in an autoclave. Finally, all specimens were subjected to slow strain rate testing at RT or 4.2 K (boiling point of liquid helium, “F” in the figure indicates the applied force). The tests at 4.2 K took place inside a liquid helium cryostat, which is surrounded by a liquid nitrogen cooled radiation shield to ensure constant specimen temperature during testing. A schematic of the test rig for the 4.2 K SSRT is shown on the right side of the image. More details are provided in Table 2.

Slow strain rate tests (SSRT) at RT were performed on a Zwick Z250 (Ulm, Germany) following EN ISO 6892-1 [36], except that the initial strain rate was set to 3 × 10^−5^ s^−1^, resulting in testing durations of ~2.5 h or less per specimen. All 4.2 K SSRTs were conducted on a Messphysik Beta 200 (Fuerstenfeld, Austria). These tests were carried out with specimens mounted inside a liquid helium cryostat surrounded by a liquid nitrogen thermal shield, ensuring specimen temperature remained at 4.2 K throughout testing. Testing commenced only after the temperature stabilization at 4.2 K. The initial strain rate used in the 4.2 K SSRTs was the same as that at RT. Strain was measured using a clip-on extensometer from the elastic region into part of the plastic regime. Due to pronounced serrated yielding at 4.2 K, the extensometer slipped predominantly in the middle of the plastic regime. Consequently, strain data beyond this point were estimated by interpolation from crosshead displacement data, based on correlation with initial extensometer strain data. This interpolation approach was validated by cross-checks against extensometer data to maintain accuracy. Final strain-to-failure values for all 4.2 K tests were determined from crosshead displacement data with an uncertainty of ±1.25% strain, ensuring consistent comparison with RT results. Two specimens were tested at RT and three at 4.2 K for each material and condition. Figure 1 schematically illustrates the described study design. The summary of the testing conditions is listed in Table 2.

Fracture surfaces were examined using a ZEISS Crossbeam 340 scanning electron microscope (SEM) at an acceleration voltage of 10 kV (Oberkochen, Germany). Microstructural characterization was performed on cross sections from the bars’ center. Samples were ground, polished and analyzed with a ZEISS GeminiSEM 450 at 5 kV (Oberkochen, Germany). To resolve nanosized precipitates, samples were additionally prepared by ion milling (Hitachi IM4000PLUS, Tokyo, Japan) and imaged at 2 kV for optimal image quality.

## 3. Results

### 3.1. Microstructure Characterization

**Figure 2:**
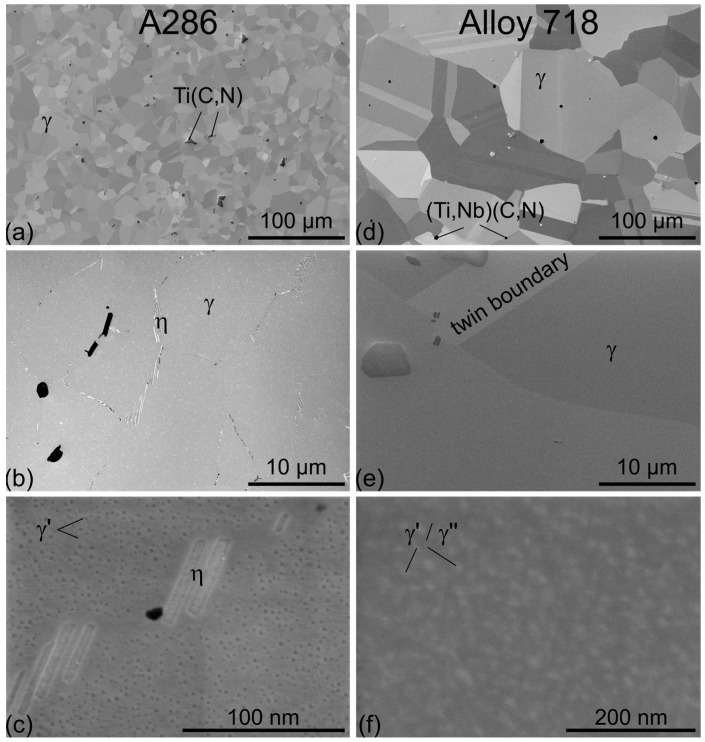
Cross-sectional micrographs of the studied materials taken from the center of the rods for A286 (**a**–**c**) and for Alloy 718 (**d**–**f**) in different magnifications: (**a**,**d**) overview of austenitic grains with carbo-nitrides, (**b**,**e**) higher magnification of austenitic grains with focus on the grain boundaries showing η phase in A286 and free grain boundaries for Alloy 718, and (**c**,**f**) high resolution of the γ′ precipitates for A286 and γ′ and γ″ precipitates for Alloy 718. Images (**a**–**d**) show secondary electron (SE) contrast, while images (**e**,**f**) show backscattered electron (BSE) contrast.

The microstructure of A286 and Alloy 718 is presented by means of SEM micrographs in Figure 2. Both alloys show the typical austenitic grains with twins and carbo-nitrides (Figure 2a,d). A286 has much finer grains than Alloy 718. A286 and Alloy 718 have average grain sizes of 13 µm (ASTM grain size number G = 9.5) and 75 µm (G = 4.5), respectively, determined according to ASTM E112-24 [37]. While the grain boundaries are free for Alloy 718 (no δ phase, Ni_3_Nb—stable phase of γ″), A286 show the typical η phase (Ni_3_Ti—stable phase of Ni_3_Ti-γ′) as white plates along some grain boundaries (compare Figure 2b,e). Both alloys are in the PH state and the precipitates are visualized in Figure 2c,f: γ′ (Ni_3_(Ti,Al)) for A286 and γ′ (Ni_3_(Al,Ti,Nb)) and γ″ (Ni_3_(Nb,Ti)) for Alloy 718. A286 does not form γ″ due to the absence of niobium. The size of the precipitates is in the range of a few nanometers. Figure 2c additionally shows a higher magnification of the η phase at a grain boundary.

### 3.2. Slow Strain Rate Tests

**Figure 3:**
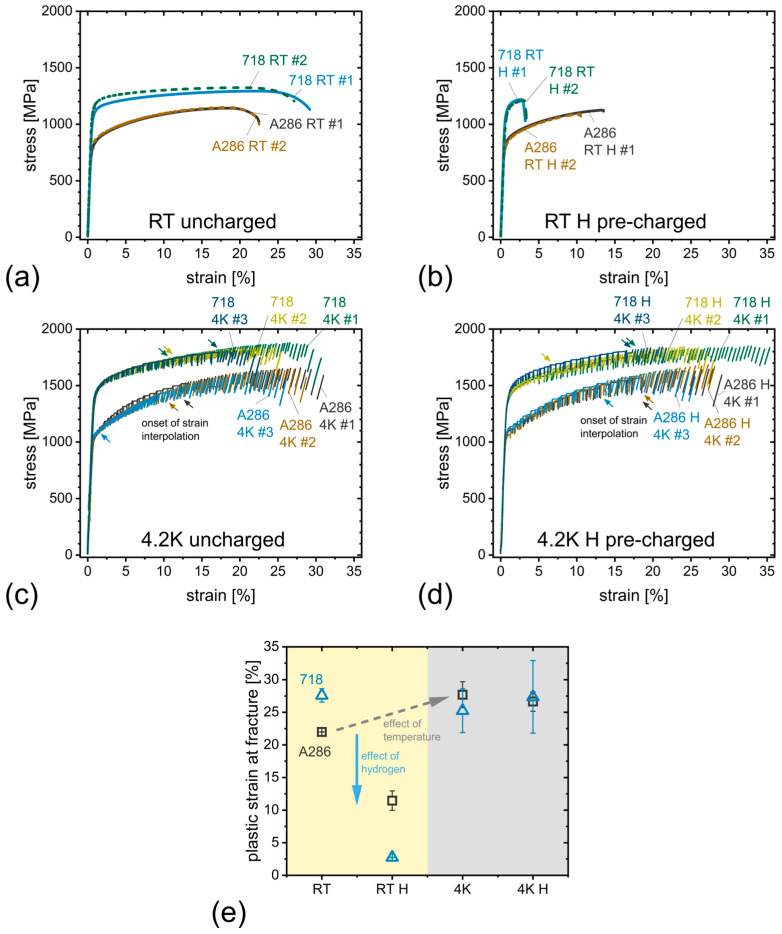
Results from SSRT at (**a**,**b**) RT, and (**c**,**d**) 4.2 K for both alloys (A286 and Alloy 718) in the uncharged and hydrogen pre-charged state, respectively. (**e**) Evaluation of the determined plastic strains at fracture for each condition and material including mean values and standard deviation. Hydrogen reduces the fracture strain at RT, whereas it remains virtually unchanged at 4.2 K. Colored arrows indicate points of strain interpolation corresponding to the respective stress–strain curves due to extensometer slip.

The results from the SSRT are shown in Figure 3. To distinguish between the RT and 4.2 K tests, as well as between the uncharged and the hydrogen-charged state, we introduce the following naming convention: We start with an indication of the alloy (“A286” or “718”) followed by the testing temperature (“RT” or “4K”) and an “H” to indicate the hydrogen pre-charged states. Finally, a number counts the specimens tested in each state, ranging from “#1” to “#3”, where two specimens per state have been tested at RT and three at 4.2 K.

RT results of A286 and Alloy 718 (Figure 3a,b) show only small differences between different specimens tested for the same state (uncharged/hydrogen-charged). Alloy 718 has a significant higher yield strength than A286 (1100 MPa vs. 800 MPa). Hydrogen pre-charged specimens show significantly lower plastic strain at fracture, a decrease from 22 to 11% for A286 and from 28 to 3% for Alloy 718. Their mean values and standard deviations are plotted in the left part of Figure 3e. The 4.2 K results show a much larger scatter of plastic strain at fracture (right part of Figure 3e), which is especially pronounced for Alloy 718. The associated stress–strain curves at 4.2 K are shown in Figure 3c,d for both alloys in its uncharged and hydrogen pre-charged conditions, respectively. All curves show serrated yielding, which is a typical phenomenon and will be analyzed later in more detail. Note that, arrows indicate the point, where strains have been interpolated from the crosshead movement, because the clip-on extensometer slipped. This causes a visible change in the zig-zag pattern of the curves.

At 4.2 K, the plastic strain at fracture has been measured as 25.3 ± 4.1% and 27.3 ± 6.8% for Alloy 718 in uncharged and hydrogen-charged conditions, respectively, which is comparable to the uncharged alloy’s RT result (27.6 ± 1.0%). A286, however, has shown an increase in plastic strain at fracture by approximately 5% with decreasing temperature from RT to 4.2 K. The plastic strain at fracture has been measured as 22.0 ±0.0% for the uncharged RT condition and 27.7 ± 2.4% and 26.6 ± 1.8% for the 4.2 K uncharged and hydrogen-charged conditions, respectively. Additionally, an increased strength has been measured with decreasing temperature in both alloys—specifically, a rise in yield strength from 800 MPa at RT to 1040 MPa at 4.2 K for A286 and from 1100 MPa at RT to 1400 MPa at 4.2 K for Alloy 718.

The plastic strain at fracture ε_f_ of hydrogen-charged and uncharged specimens have been compared using the ductility loss, as defined in Equation (1):

(1) $$ductility\;loss\left[\%\right]=\left(1-\frac{\varepsilon_{f\;charged}}{\varepsilon_{f\;uncharged}}\right)\times 100.$$

A286 and Alloy 718 exhibited ductility losses of 48% and 90% at RT, respectively, which are attributed to the substantial hydrogen uptake of 89 and 107 wt. ppm.

For the 4.2 K tests, no significant influence of hydrogen charging on plastic strain at fracture or the stress strain curve behavior has been observed in both materials.

**Figure 4:**
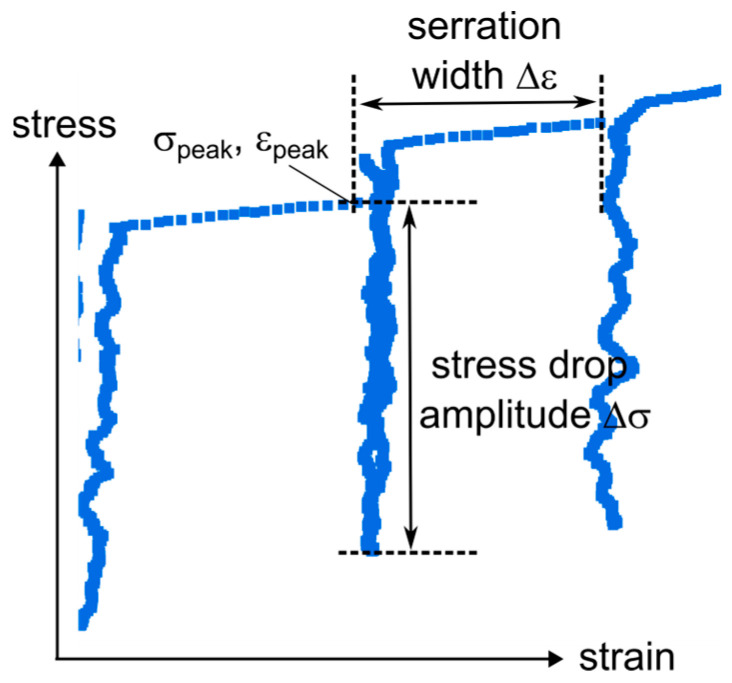
Schematic illustration of serrations at 4.2 K. Definition of stress drop amplitude (Δσ = σ_peak_ − σ_valley_) and serration width (Δε).

Figure 4 shows a schematic illustration of the serration and illustrates the definition of the stress drop amplitude (Δσ = σ_peak_ − σ_valley_) and the serration width (Δε, defined as the strain difference between a serration peak and the preceding peak). The serration analysis at 4.2 K was conducted with the following considerations. First, the three datasets per state (uncharged and hydrogen-charged for each alloy) were treated equally, resulting in four combined datasets: A286 4K, A286 4K H, 718 4K and 718 4K H. Second, only serrations measured by the clip-on extensometer were analyzed. This restricted the analysis to strains up to 10% and excluded A286 4K #3 and 718 4K H #2 due to early extensometer slip.

**Figure 5:**
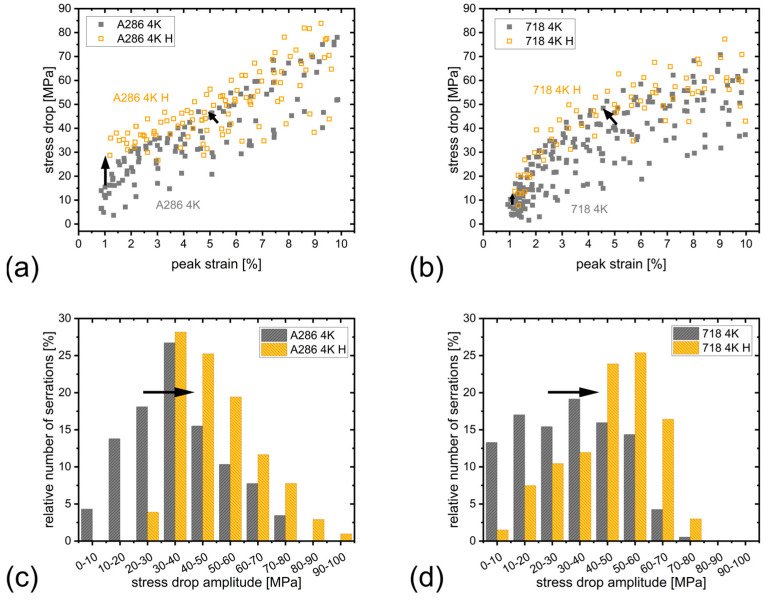
Results from the serration analysis up to 10% strains for A286 (**a**,**c**) and Alloy 718 (**b**,**d**): (**a**,**b**) stress drop amplitude (Δσ) as a function of the peak strain (ε_peak_), and (**c**,**d**) histogram of the stress drop amplitude. Hydrogen pre-charged states show higher stress drop amplitudes, as indicated by the black arrows.

The results are presented in Figure 5 for A286 (left: a, c) and Alloy 718 (right: b, d). The stress drop amplitude (Δσ) as a function of peak strain (Figure 5a,b) shows a distinct influence of hydrogen charging. Although the data exhibit considerable scatter, the hydrogen-charged state consistently displays higher stress drop amplitudes in both alloys, as highlighted by the black arrows. A comparison of the hydrogen-charged states further reveals that the first stress drops are significantly larger in A286 than in Alloy 718. The distribution of the stress drop amplitudes (Δσ) is shown in Figure 5c,d. Both alloys exhibit higher Δσ in the hydrogen-charged state compared to the uncharged state.

Table 3 summarizes the dataset counts and number of serrations, together with the calculated serration frequency, mean stress drop amplitude Δσ and mean serration width Δε. Both alloys show increased mean Δσ and Δε in the hydrogen-charged state. This is accompanied by a reduction in serration frequency per percent strain.

### 3.3. Fracture Surfaces

**Figure 6:**
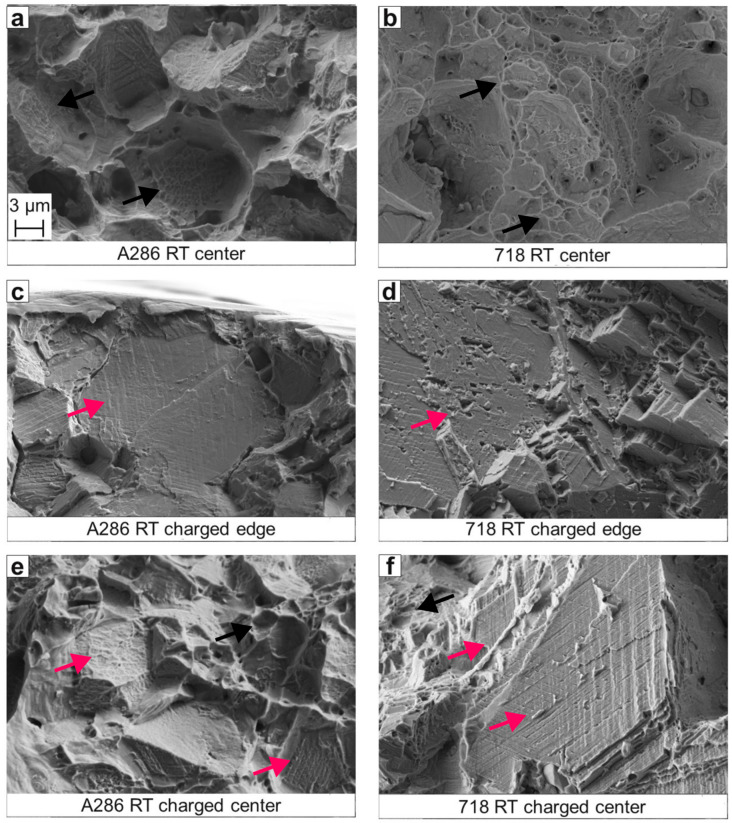
Representative SEM fractographs of tensile A286 specimens measured in the (**a**) center of the fractured specimen surface tested at RT without charging and at the (**c**) edge and in the center (**e**) of the fractured specimen surface tested at RT after hydrogen charging. (**b**,**d**,**f**) The same measurements performed for Alloy 718. Black arrows indicate ductile dimples, while magenta arrows highlight brittle cleavage features. All images show secondary electron (SE) contrast.

The SEM fracture surface analysis of specimens after SSRT at RT is presented in Figure 6. Specimens not subjected to hydrogen charging exhibit ductile dimples at the fracture surface center, surrounded by shear fracture region (Figure 6a,b). Conversely, hydrogen-charged specimens tested at RT (Figure 6c–f) display brittle failure at the edges, where fracture has been initiated, and a mix of brittle (cleavage and partly intergranular) and ductile (dimples) failure modes for Alloy 718 in the center, while A286 shows predominantly ductile dimples.

**Figure 7:**
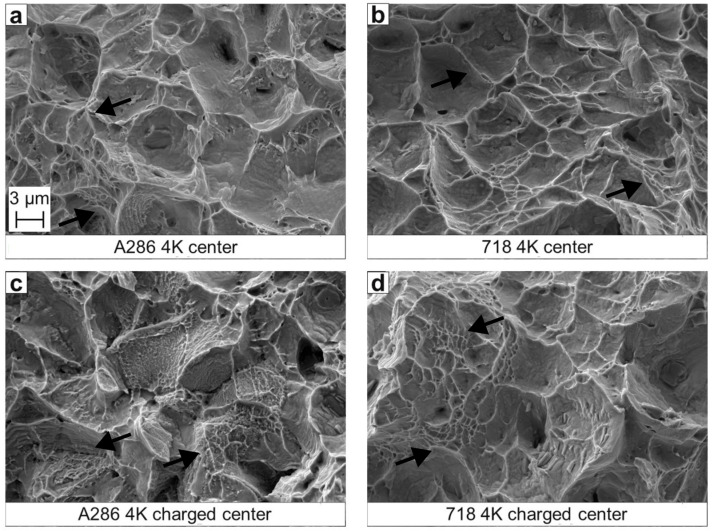
Representative SEM fractographs of tensile specimen tested at 4.2 K: (**a**,**c**) refer to A286 tested under uncharged and hydrogen charged conditions, respectively; (**b**,**d**) refer to Alloy 718 tested under uncharged and hydrogen charged conditions, respectively. Black arrows indicate ductile dimples. All images show secondary electron (SE) contrast.

The SEM analysis of fracture surfaces from SSRT at 4.2 K is presented in Figure 7. All specimens exhibit a fully ductile center characterized by dimples, encircled by shear fracture regions. Notably, no pronounced difference is observed between hydrogen-charged and uncharged conditions.

## 4. Discussion

### 4.1. Ductility Loss at RT and Liquid Helium Temperature

The significant ductility losses observed at RT, approximately 48% for A286 and 90% for Alloy 718, align well with previously reported reduction in area measurements from ex situ tensile tests under comparable hydrogen charging conditions [3,5,13]. Both A286 and Alloy 718 have been recognized for their good resistance against hydrogen embrittlement [2].However, ductility loss strongly depends on hydrogen concentration [38]. In this study, the pronounced ductility losses correlate with substantial hydrogen uptake of 89 wt. ppm for A286 and 107 wt. ppm for Alloy 718.

The difference in the ductility loss magnitude between the two alloys can likely be explained by their variations in yield strength and microstructure. At RT, Alloy 718 exhibits a higher yield strength of approximately 1100 MPa compared to around 800 MPa for A286, mainly due to the additional hardening phase, γ″, alongside the γ′ phase present in both alloys. Hydrogen embrittlement susceptibility typically increases with strength [39] and with the volume fraction of γ′ and γ″ precipitates [9], although disentangling these effects remains challenging. Density functional theory (DFT) studies suggest hydrogen segregation at the matrix-γ″ interface reduces the strain to fracture, suggesting a possible role of these precipitates in hydrogen-induced ductility loss [40]. Furthermore, matrix composition, particularly austenite stability reflected in nickel equivalent [41], strongly influences hydrogen embrittlement resistance. Omura et al. [42] and Lee and Woods [11] reported that increased nickel content raises hydrogen embrittlement sensitivity, especially after precipitation hardening, consistent with Alloy 718 being more susceptible than A286.

Despite the pronounced ductility loss in hydrogen-charged specimens at RT, the uncharged A286 alloy exhibits increased ductility and strength when tested at 4.2 K. This improvement primarily stems from its low SFE, which is well known to enhance ductility in austenitic stainless steels at cryogenic temperatures [26,27,28]. The SFE decreases further with temperature, promoting deformation mechanisms such as twinning and increased strain-hardening rates [43]. Additionally, low SFE restricts cross slip and dislocation climb, contributing to the cryogenic strengthening observed in both A286 and Alloy 718.

### 4.2. Low Temperature Serrated Deformation

Considering deformation mechanisms at cryogenic temperatures, characteristic serrated flow behavior warrants special attention. LTSD has been reported across various metals and alloys under these conditions [24,26,27,44,45,46,47]. Obst and Nyilas [46] proposed that LTSD originates from planar dislocations (edge dislocations) piling up against intragranular obstacles, such as Lomer–Cottrell (LC) locks [48]. As applied stress increases, it reaches the cohesive strength of these obstacles, causing their collapse. This collapse can propagate through neighboring dislocation pile-ups, triggering avalanche-like dislocation motion and resulting in the distinctive stress drops observed.

LTSD may arise from twinning, characterized by disordered serration amplitudes, or from plastic slip instabilities, where the serration amplitude increases monotonically with deformation [24]. The latter behavior is observed here for A286 and Alloy 718 (Figure 5a,d), attributing serrations to localized plastic deformation from dislocation accumulation at obstacles. In austenitic alloys, martensitic transformations can also cause serrations, complicating distinction from plastic slip instability [24]. However, Fe-Cr-Ni alloys with 20 to 25% nickel and 18% chromium do not undergo phase transformations [24,49]. Given A286’s composition of approximately 25% nickel and 15% chromium, phase transformations are not expected and thus unlikely to influence serration behavior.

The impact of precipitates, namely γ′ and γ″, on LTSD remains largely unexplored. Molecular dynamics simulations have shown that at low temperatures edge dislocations traverse Ni_3_Al precipitates without forming dislocation locks [50]. This is consistent with the view that LC locks predominantly form within the matrix rather than at precipitate interfaces. The LTSD may also be attributed in some cases to local thermal softening effects caused by short-term temperature fluctuations and temperature dependence of thermal conductivity (thermo-mechanical effect) [24]. In the case of A286 and Alloy 718, the observed LTSD effect can most likely be attributed to the accumulation of dislocations at obstacles such as LC locks. Following the results of our DFT exact muffin-tin orbitals (EMTO) calculations (fcc Fe_63_Cr_15_Ni_22_ and fcc Fe_21_Cr_22_Ni_57_, paramagnetic at 300K), both alloys exhibit SFE of 24 mJ/m^2^ and in 30 mJ/m^2^, respectively, which is very close to that reported for an austenitic alloy with the SFE of 30 mJ/m^2^ in Ref. [29], where dislocation pile-ups at LC locks were directly imaged using transmission electron microscopy (TEM). The compositions used as input for the EMTO calculations represent simplified matrix composition of A286 and Alloy 718, as given in Ref. [51] and Ref. [52], respectively. The details of our DFT EMTO calculations are identical to those in Ref. [53].

### 4.3. Effect of Hydrogen on Serrations at Liquid Helium Temperature

Analysis of serrations up to 10% strain reveals notable differences for hydrogen pre-charged specimens. Specifically, the mean stress drop amplitude (Δσ) increases by a factor of 1.4 for both A286 and Alloy 718. Since stress drop amplitude correlates with the number of dislocations ahead of LC locks [24,26], these results suggest that hydrogen promotes greater dislocation pile-ups. As serrations reflect inhomogeneous deformation, hydrogen enhances this localized behavior. The increased dislocation pile-ups relate to the pinning force of LC locks. Stress drops, as avalanche-like events, may arise from multiple LC locks, implying that higher pinning forces arise either from increased LC lock density due to greater dislocation density or from increased pinning strength per lock caused by hydrogen.

The former requires increased dislocation density, which would manifest as changes in strain hardening; however, no such changes are evident in the tensile curves. The latter, increased pinning strength, ties to the lock’s cohesive energy [46], that is, the energy needed to separate the sessile dislocation into its partial dislocations. The SFE governs the separation of partial dislocations and thereby the strength of LC locks; an increase in SFE leads to stronger locks [54]. Since hydrogen is known to reduce SFE [55,56], one would expect a reduction in lock strength, contrary to our observations of increased stress drop amplitudes.

Stumpf et al. [57] observed an increased LC lock density due to carbon addition in an austenitic Cr-Co-Ni alloy. Hydrogen might induce a similar effect, as both, hydrogen and carbon are interstitial elements. We therefore hypothesize that hydrogen’s reduction in SFE alters the probability of two leading partial dislocations forming sessile locks, increasing their number probabilistically. Hence, hydrogen increases stress drop amplitudes primarily by raising lock density rather than individual lock strength.

Alongside larger stress drops, we observe increased strain accumulation during serrations (serration width, Δε) by factors of 1.6 and 1.5 in hydrogen-charged A286 and Alloy 718, respectively. This aligns with increased dislocation pile-up inferred from stress drop data. Correspondingly, serration frequency, that is, number of serrations per percent strain, decreases by factors of 0.6 and 0.5 for A286 and Alloy 718, respectively.

These results indicate that hydrogen interacts with dislocations even under cryogenic conditions. However, no embrittlement was detected in either alloy, consistent with Ogawa et al.’s [13] findings that Alloy 718’s hydrogen-induced ductility loss decreases markedly below −80 °C despite significant hydrogen uptake. In their work, embrittlement was attributed to diffusion-mediated mechanisms such as hydrogen condensation near dislocations and microcrack nucleation along annealing twin boundaries or {111} slip planes.

At 4.2 K, hydrogen mobility is strongly suppressed, preventing diffusion-assisted embrittlement processes. Instead, only localized hydrogen–dislocation interactions manifest as altered serration behavior. Although hydrogen is essentially immobile at this temperature, it may still influence dislocation activity through its presence in lattice traps such as dislocations, interfaces, or precipitates. In this trapped state, hydrogen can slightly modify local strain and stress fields, thereby affecting the accumulation and interaction of dislocations with obstacles such as LC locks. Though similar trends were observed in both alloys, microstructural differences may influence precise serrated yielding mechanisms. Nonetheless, the consistent absence of embrittlement suggests diffusion limitations play a dominant role over alloy-specific deformation mechanisms in fracture behavior at cryogenic temperatures.

## 5. Conclusions

This study demonstrates that gaseous hydrogen charging at 473 K and 1000 bar for 14 days causes significant hydrogen uptake of 89 wt. ppm in A286 and 107 wt. ppm in Alloy 718, which results in substantial ductility loss at room temperature during slow strain rate tensile testing at an initial strain rate of 3 × 10^−5^ s^−1^. Alloy 718 exhibits a drastic 90% reduction in ductility, while A286 shows a 48% loss. These large ductility losses occur despite both alloys being generally regarded as hydrogen resistant, highlighting the critical role of hydrogen concentration in embrittlement under these aggressive charging conditions.

At liquid helium temperature (4.2 K), no measurable ductility loss due to hydrogen charging is observed in either alloy, indicating that hydrogen is effectively immobile under cryogenic conditions. Both uncharged and hydrogen pre-charged specimens exhibit serrated flow behavior during tensile testing at 4.2 K. However, hydrogen modifies this behavior by increasing the stress drop amplitudes and strain accumulation within serration events. These alterations suggest that hydrogen–dislocation interactions might remain active at cryogenic temperatures, enhancing dislocation pinning forces by obstacles such as Lomer–Cottrell locks.

Both alloys exhibit significantly increased yield strengths at 4.2 K, with A286 showing improved ductility (~22% to ~27%), while Alloy 718 maintains similar ductility levels. These results suggest that, while hydrogen influences plasticity mechanisms at cryogenic temperatures, hydrogen embrittlement is suppressed, making both alloys promising candidates for hydrogen service in cryogenic environments.

## Figures and Tables

**Table 1:** Nominal composition of A286 and Alloy 718 in wt.%.

Alloy	UNS Number	Al	C	Cr	Fe	Mo	Nb	Ni	Ti
A286	S66286	≤0.35	≤0.08	13.5–16.0	bal.	1.0–1.5	-	24–27	1.90–2.35
Alloy 718	N07718	0.5	0.01	18	18	3	5	bal.	1

**Table 2:** Summary of the testing conditions.

	SSRT at RT (3 × 10^−5^ s^−1^)	SSRT at 4.2 K (3 × 10^−5^ s^−1^)
uncharged	A286 RT (#1, #2) 718 RT (#1, #2)	A286 4K (#1, #2, #3) 718 4K (#1, #2, #3)
H_2_ 1000 bar, 473 K, 14 days (pre-charged)	A286 RT H (#1, #2) 718 RT H (#1, #2)	A286 4K H (#1, #2, #3) 718 4K H (#1, #2, #3)

**Table 3:** Serration analysis up to 10% strain in A286 and Alloy 718 (uncharged vs. hydrogen-charged) at 4.2 K, reporting dataset count, serration number and frequency, mean stress-drop amplitude Δσ (MPa) and mean serration width Δε (% strain, defined as the strain difference between a serration peak and the preceding peak).

Alloy/State	Datasets (n)	Serrations (n)	Frequency (Events/% Strain)	Δσ (MPa)	Δε (% Strain)
A286 4K	2	116	5.8	37	0.16
A286 4K H	3	103	3.4	50	0.25
718 4K	3	188	6.3	32	0.14
718 4K H	2	67	3.4	46	0.21

## Data Availability

The raw data supporting the conclusions of this article will be made available by the authors on request.

## Abbreviations

The following abbreviations are used in this manuscript:

PH	Precipitation hardened
RT	Room temperature
LTSD	Low temperature serrated deformation
SFE	Stacking fault energy
SSRT	Slow strain rate tests
SEM	Scanning electron microscopy
LC	Lomer–Cottrell
DFT	Density functional theory
EMTO	Exact muffin-tin orbitals
TEM	Transmission electron microscopy
SE	Secondary electron
BSE	Backscattered electron
